# DNA Methylation Is Dispensable for Suppression of the *Agouti viable yellow* Controlling Element in Murine Embryonic Stem Cells

**DOI:** 10.1371/journal.pone.0107355

**Published:** 2014-09-05

**Authors:** Athanasia Stathopoulou, Giulia Lucchiari, Steen K. T. Ooi

**Affiliations:** Department of Cancer Biology, University College London Cancer Institute, London, United Kingdom; Peking University Health Science Center, China

## Abstract

The *agouti viable* (*A^vy^*) locus is considered a model to understand how retroelements function as controlling elements in mammals. Epigenetic factors, principally CpG methylation, are widely held to play a dominant regulatory role in controlling the locus' activity. The purpose of this study was to examine its behavior in ES cells and determine if this locus could be exploited for use in screen-based investigations. We have derived multiple *A^vy^* ES cell lines from the C57BL/6 strain and generated a cell line carrying a GFP-reporter gene (*A^vy^*/*A^GFP^*). Use of the DNA demethylating drug 5-azacitidine on various ES cell lines does not induce either *agouti* or GFP expression. Methylation analysis reveals that although most lines display normal methylation at IAP elements in general, the *A^vy^* IAP element is essentially unmethylated. In addition, we find that different repeat compartments are epigenetically unstable in a number of derived cell lines.

## Introduction

Retrotransposons constitute a large proportion of the mammalian genome and pose a significant threat to its integrity [1]. Although most retroelements are inactive, a large number remain active and are capable of interfering with transcription of genes located nearby, serving as ‘controlling elements’ [2]. This interference is mediated by CpG-dense 5′ and 3′ sequences, which include endogenous retrovirus (ERV)-specific long terminal repeats (LTRs) and can serve as potent promoters/enhancers [3], [4]. Although predominantly silent in somatic/differentiated cells, retrotransposons are thought to be most active during two stages of organismal development; during gametogenesis [5] and in the early embryo [6]. Repression is mediated by multiple factors including KRAB zinc finger proteins (KRAB ZFP) [7], KAP1 [8] and piRNAs [9]. Epigenetic mechanisms play a dominant role in their silencing; indeed, one of the major functions of DNA methylation in mammals is to repress retroelements [10], [11]. The histone methyltransferase Setdb1 also plays a crucial role in their silencing [12]. However, our understanding of the mechanism by which these various factors interact to mediate repression remains incomplete.

The agouti viable yellow (*A^vy^*) mouse strain is one of the best-studied examples of how a retroelement can interfere with neighbouring gene expression resulting in a phenotypic consequence. Over 50 years ago, the allele arose spontaneously as a result of an IΔ1-class intracisternal A particle (IAP) retrotransposon inserting into an upstream non-coding exon (pseudoexon 1A) of the *agouti* gene, located about 100 kb from the first coding exon [13], [14]. When the IAP is active, transcription from a cryptic promoter in the 3′LTR drives ectopic *agouti* expression [15]. Constitutive expression in all tissues results in obese yellow animals; silencing of the IAP permits correct *agouti* expression and such animals are termed pseudoagouti. The IAP is subject to epigenetic regulation and the activity state of the *A^vy^* allele is inversely correlated with DNA methylation at the IAP element [16].

To date, all studies on the role of DNA methylation at the *A^vy^* IAP have focused on adult animals and tissues. Here we describe the first study examining DNA methylation in embryonic stem (ES) cells derived from *A^vy^* animals and their utility.

## Materials and Methods

### Derivation of *A^vy^* ES cells

Timed matings were set up between male and female *A^vy^* animals (B6.C3-Avy/J, Jackson Labs). To increase the number of embryos, female animals received injections to stimulate superovulation (gonadotrophin from pregnant mare serum) followed by human chorionic gonadotrophin. 3 days after detection of a copulation plug, females were sacrificed and uterine horns dissected out. Embryos were flushed out and collected before being individually seeded onto mitomycin-C treated mouse embryonic fibroblasts in standard FBS-containing ES cell media supplemented with 2i (PD03259010 and CHIR99021) in 96-well plate format. 2i was only included during the initial derivation phase. Six days after initial seeding, wells were tryplated onto MMCT-MEFs and passaged every third day. Established ES cell clones were checked by morphology and expanded up for further experiments. All animals used for this study were covered by a Home Office Project License under The Animals (Scientific Procedures) Act 1986 to S.K.T.O. In addition, this research study was approved by the UCL Research Ethics Committee. Mice were humanely sacrificed at a designated establishment by cervical dislocation.

### Generation of *A^vy^/A^GFP^* ES cells

**Figure 1:**
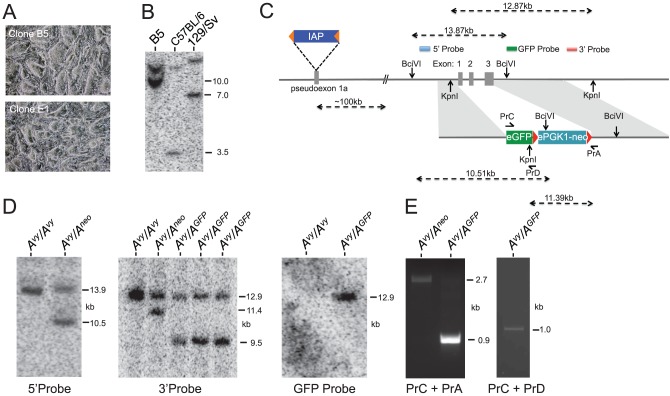
Generation of *A^vy^-GFP* ES cells. **A.** Representative images of ES cells derived from the Avy strain. Clone B5 was used for subsequent gene targeting experiments. **B**. Confirmation that clone B5 is homozygous for the Avy allele. For comparison of the different alleles, DNA from C57BL/6 and 129/Sv animals was also probed. The *A^vy^* allele is indicated by a 10 kb fragment. The *α* allele (present in the C57BL/6 strain) is 3.5 kb whilst the *A* allele (present in the 129/Sv strain) is 7.0 kb. A non-specific and strain-independent 11 kb band is also observed. **C.** Targeting strategy to replace the *agouti* locus with an *eGFP* reporter cassette. **D.** Southern blot data confirming gene targeting using the restriction strategy and probes as indicated in **C**. DNAs were digested with the following enzymes: *Bci*VI (5′ Probe), *Kpn*I (3′ and GFP Probe). **E.** PCR strategy and results indicating presence of the *eGFP* cassette in targeted cell lines. Prior to Cre-mediated removal of the *neo* cassette PrC and PrA generates a 2,704 bp amplicon; after removal the amplicon is 907 bp in size. PrC and Pr D are specific for the eGFP cassette and generate a 1,046 bp amplicon.

The *A^vy^-GFP* replacement construct was generated using a bacterial artificial chromosome (BAC) (RP23-466H11) containing approximately 180 kb of mouse chromosome 2 encompassing the *agouti* locus (CHORI). A 17.4 kb fragment of this was retrieved into the vector pL253 by BAC recombineering in the recombineering strain SW102 [17]. This was further targeted with a PCR amplified eGFP construct (pEGFP-C1, Clontech) containing homology arms flanking the *agouti* locus. For the eGFP cassette, pEGFP-C1 (Clontech) was used as the template and PCR-amplified using primers spanning the Start ATG and SV40 early mRNA Polyadenylation Signal. The replacement construct was sequence verified before being used to target one of the *A^vy^* ES cell lines derived (clone B5). Targeting was performed as previously described [18]. Following identification of a correctly targeted clone (*A^vy^-neo*), this was further expanded and the *neo* selection cassette was removed by transient transfection using the Cre expression plasmid pCAGGS-Cre [19]. Clones deleted for the *neo* cassette (*A^vy^/A^GFP^*) were expanded and used in the experiments described. Primer sequences (5′ to 3′) used to generate the amplicons shown in Figure 1E: TTG GAG GCA GCC TAG ACA CT (Primer A); TTG GAG GCA GCC TAG ACA CT (Primer C); GAA TTC CTC TAA GAT ACA TTG ATG AGT (Primer D). Primer sequences used to sex derived ES cell lines: AGA GAT CAG CAA GCA GCT GG (*sry*_F); TCT TGC CTG TAT GTG ATG GC (*sry*_R).

### Southern Blot

Kit extracted DNA (GeneJET Genomic DNA Purification Kit, Thermo Scientific) was digested using the appropriate enzyme. 10 µg and 1 µg of DNA was digested for genotyping and methylation analysis Southern blots, respectively, for 6 hours. Digested DNAs were resolved on 0.8% TAE gels by electrophoresis before image capture. Gels were then depurinated (0.2M HCl, 15 minutes), denatured (0.5M NaOH, 1.5M NaCl, two washes, 30 minutes each) and neutralized (1M Tris pH 8.0, 1.5M NaCl, two washes, 30 minutes each) before blotting onto a 0.45 mm Nylon membrane (Nytran, GE) overnight. The next day, transferred DNA was fixed by UV cross-linking then incubated in Prehybridization Solution (6× SSC, 5× Denhardts, 1% SDS, 10% Dextran Sulphate) for at least two hours before overnight incubation with radiolabelled probes. For methylation analysis, probes used were described previously [20], [21]. For *agouti* genotyping, the probe used is as described in Duhl et al. [15].

### RNA extraction and quantitative real-time PCR (qRT-PCR)

Total RNA was extracted using TRIZOL reagent according to the manufacturer's protocol (Life Technologies). Prior to use for PCR, RNAs were DNase I treated (TURBO DNase, ambion) to remove any residual contaminating DNA. Expression of the different sequences indicated was determined using the Roche Universal Probe Library System (Roche Diagnostics). To detect IAP, a suitable hydrolysis probe was generated using a full-length consensus IAP sequence (GenBank accession number M17551). Primer sequences (5′ to 3′) used: TGA CAG GAG TCT GCG GAG TA (qAgouti_F); TCT TGG ATT TCT TGT TCA GTG C (qAgouti_R); TTG GCA GAT AAG GCC ACC TA (qIAP_F); AAT TTC TTG GGC AGC CTC TAC (qIAP_R); TCG TGA CCA CCC TGA CCT AC (qGFP_F); AAG TCG TGC TGC TTC ATG TG (qGFP_R). UPL probes and reference genes used: Agouti (UPL Probe 82, GAPDH), IAP (UPL Probe 32, GAPDH), GFP (UPL Probe 41, GAPDH).

### Western Blot

RIPA lysates were prepared from pelleted cells. Following lysis on ice, and centrifugation to clarify, samples were quantified and diluted into 1× Laemmli Buffer. Approximately 50 µg of lysate was loaded in each lane of a 10% Mini-Protean TGX Gel (Bio-rad). Following separation by electrophoresis, proteins were transferred onto PVDF membrane. Membranes were then blocked in 1× PBS+0.1% Tween-20 (PBST)+5% milk for one hour before incubating with anti-GFP antibody (1∶2,000, Rabbit, polyclonal, A-1122, Life technologies) overnight. The next day, membranes were washed in PBST (6 times, 5 minutes), before incubation with secondary antibody (1∶5,000, donkey anti-rabbit HRP, NA934V, GE Healthcare) for one hour at room temperature. Membranes were then washed in PBST (6 times, 5 minutes), before HRP signal was developed using Luminata Forte Western HRP Substrate (Millipore). Signals were detected by exposure onto Hyperfilm ECL (Amersham). Post-signal acquisition, membranes were washed in PSBT and re-probed using anti-tubulin antibody (1∶10,000, mouse monoclonal, T6199, Sigma, followed by donkey anti-mouse HRP, 1∶5,000) using the same methods as described.

### Bisulphite conversion and sequencing

Kit-purified DNA was bisulphite converted using the EZ DNA Methylation Lightning Kit (Zymo Research) according to the manufacturer's protocol. Cloning and analysis were performed as previously described [22]. Primer sequences (5′ to 3′) used to detect the Avy IAP LTR element were: GTA GAG GTT TAA GGA TTT AGA TTG GTG (Avy bis fwd); AAC CCA CAA AAC CAA AAT CTT CTA C (Avy bis rev).

### 5-Aza and TSA Treatment

Prior to the start of drug treatment, ES cells were seeded and grown in culture for 42 hours. Drug-containing media was then added to cells and the cells left for a further 30 hours before harvesting for analysis.

## Results and Discussion

The paradigm of the *A^vy^* locus as a ‘controlling element’ is that the associated IAP element aberrantly controls *agouti* expression. From the existing data in animals/differentiated cells, the *A^vy^* IAP is predicted to be regulated by DNA methylation at the 5′ LTR sequence. We therefore reasoned that this would be an ideal system from which to generate a GFP-based reporter cell line for use in screen-based approaches to identify novel regulators necessary for retrotransposon silencing. To that end, we derived ES cells from animals where the *A^vy^* IAP element has been inbred onto the C57BL/6 background. Images of representative derived lines are shown in Figure 1A. Genetic analysis confirmed that these cells were homozygous for the *A^vy^* allele (Figure 1B). The homozygousity of the *A^vy^* allele was important as this ensured that the GFP cassette would be located downstream of the inserted IAP element, irrespective of which copy of the *agouti* gene that was substituted/targeted. A non-specific and strain-independent background band was also observed; this is consistent with the original report describing this genotyping strategy [15]. One cell line (clone B5) was subjected to gene targeting by homologous recombination using an *eGFP* cassette designed to replace the endogenous *agouti* locus. Figure 1C outlines the targeting strategy adopted. Southern blot analysis using 5′-, 3′- and GFP-specific probes indicated successful targeting of this locus (Figure 1D). PCR at the targeted locus generated amplicons at the expected sizes both before and after Cre-mediated removal of the floxed *neo* selection cassette (Figure 1E).

**Figure 2:**
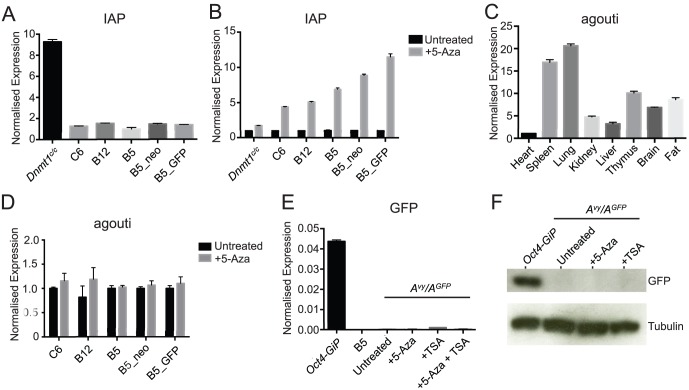
Analysis of IAP and *agouti* expression in *A^vy^* ES cells. For qRT-PCR analyses, bar graphs represent mean+/− S.D. Results are representative of two independent experiments. **A.** qRT-PCR analysis of IAP expression. Expression levels are normalized to actin. RNA from *Dnmt1^c/c^* ES cells was used as a positive control for IAP expression. **B.** qRT-PCR analysis of *IAP* expression in cells either untreated or treated with the DNA demethylating agent 5-azacytidine (5-aza, 7.5 µM). Expression levels are normalized to actin and expressed as fold change relative to the signal observed in untreated cells. **C.** qRT-PCR analysis of *agouti* expression in the various organs indicated. Expression levels are normalized to *GAPDH* and measured relative to expression level in heart. **D.** qRT-PCR analysis of *agouti* expression in cells either untreated or treated with the DNA demethylating agent 5-aza. Expression levels are normalized to GAPDH and measured relative to untreated signals. **E.** qRT-PCR analysis of *GFP* expression in the various cell lines indicated. RNA from *Oct4-GiP* ES cells was used as a positive control for GFP expression. Expression levels are normalized to actin. **F.** Western blot data using an anti-GFP antibody on whole cell lysates from *A^vy^/A^GFP^* ES cells either untreated or treated with 5-aza or the histone demethylating agent Tricostatin A (TSA, 40 nM). Whole cell lysate from *Oct4-GiP* ES cells was used as a positive control. Western blot using an anti-tubulin antibody was used as a loading control.

We then assessed the ability of these cells to report on IAP activity. DNA methylation at CpG-dense LTR sequences is known to control expression of IAP elements. ES cells deficient for the maintenance DNA methyltransferase *Dnmt1* show abundant transcription of IAP elements [20]. Importantly, silencing of IAPs is dependent on the methyltransferase activity of Dnmt1. Consistent with this, IAP expression could only be detected in *Dnmt1^c/c^* ES cells; none of the *A^vy^* ES cells examined showed signs of IAP expression under normal culture conditions (Figure 2A). However, IAP expression could be induced by treatment with the DNA demethylating drug 5-azacitidine (5-aza) (Figure 2B). Interestingly, 5-aza induced IAP expression was lowest in *Dnmt1^c/c^* ES cells.

Under the prevailing model and combined with our observations we predicted that loss of DNA methylation at IAP elements, including the *A^vy^* IAP element, should result in *agouti* gene expression. Consistent with previous studies [15], using RNA extracted from various organs from a yellow *A^vy^* animal (indicative of pan-cellular expression of agouti), we detected robust and ectopic *agouti* expression by qRT-PCR (Figure 2C). However, 5-aza-treatment of *A^vy^* ES cells did not induce *agouti* expression (Figure 2D). This was also the case for GFP expression using our targeted reporter cell line. Although we could detect GFP expression using whole cell lysates extracted from a GFP expressing ES cell line (*Oct4-GiP*), we were unable to induce GFP expression in our targeted reporter cell line using either 5-aza or the histone deacetylase inhibitor Trichostatin A (TSA) (Figure 2E and 2F). Therefore, despite extensive literature correlating DNA methylation levels at the *A^vy^* IAP element and the regulation of the *agouti* gene in differentiated cells, our data demonstrate this is uncoupled in pluripotent stem cells. This also indicates that other mechanisms must be involved in the control of this locus. In this regard, it is interesting that transcriptome analyses of ES cells deficient for *Setdb1*, which mediates trimethylation of lysine at position 9 of histone H3 (H3K9me3), suggest it plays a major role in silencing multiple classes of retroelements including IAPs [23].

**Figure 3:**
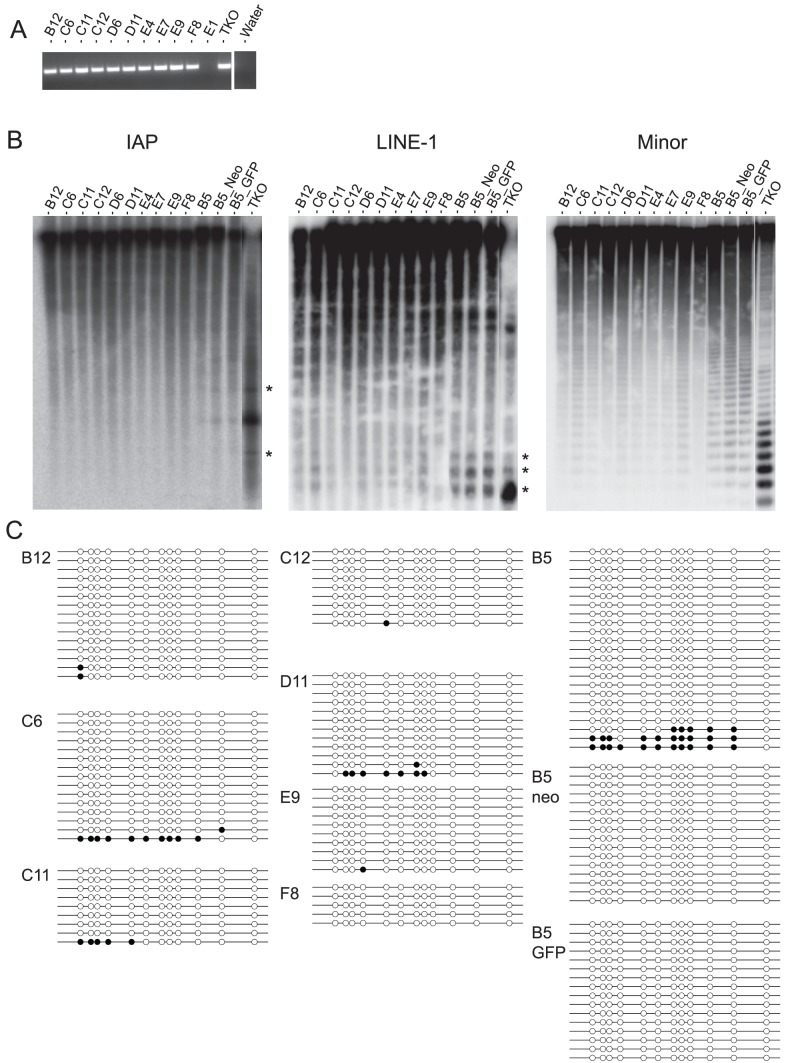
Methylation analysis of *A^vy^* ES cell lines. **A.** *Sry* PCR genotyping result of the various cell lines used in this study. PCR on DNA from XY ES cells generate generates a 249 bp amplicon. Clone E1 is *Sry* ^−^ and inferred to be XX. **B.** Methylation sensitive Southern blot data on DNA extracted from the various *A^vy^* ES cell lines indicated. DNA extracted from *Dnmt1^−/−^Dnmt3a^−/−^Dnmt3b^−/−^* (TKO) ES cells was included as a control for unmethylated signal. Asterisks indicate hypomethylated/unmethylated fragments. DNAs were digested with *Hpa*II restriction endonuclease. Probes used are as indicated. **C.** Bisulphite sequencing data using primers specific for the *A^vy^* IAP LTR on DNA from the *A^vy^* cell lines indicated. Passage numbers of *A^vy^* ES cells are as follows: passage 6 (C12 and F8), passage 7 (B12, C11, D6, D11, E4, E7 and E9); passage 10 (C6), passage 25 (B5), passage 35 (B5_Neo), passage 45 (B5_GFP).

We next sought to understand why drug-mediated inhibition of DNA methylation failed to induce both *agouti* and GFP expression. To that end, we examined the methylation status within the repeat compartment of eleven different *A^vy^* ES cell lines that were derived. Primers designed to detect the presence of the *Sry* locus by PCR confirmed that all the cell lines analysed were *Sry* ^+^ and therefore XY (Figure 3A). We performed methylation sensitive Southern blots probing for IAP, Long Interspersed Nuclear Element-1 (LINE1-1) and minor satellite elements. For all three repeat compartments, hypomethylation was observed in clone B5 (passage 25) and its derivative, targeted sub-clones (B5_Neo (passage 35) and B5_GFP (passage 45) (Figure 3B). However, there was more variability observed with the other cell lines examined. At the resolution afforded by methylation sensitive Southern blot, IAP elements were found to be normally methylated in all other cell lines, but this was not the case for LINE-1 and minor satellite sequences. Clones C6 (passage 10), D6 and E9 (both passage 7) all showed signs of hypomethylation at these sequences. A recent study has documented differential methylation at IAP and Early Transposon/Mus musculus type D (ETn/MusD) classes of ERVs located close to gene transcription start sites (TSS) in C2 ES cells, a C57BL/6-derived cell line [24]. To the best of our knowledge, our results are the first description of DNA methylation in the LINE-1 and minor satellite repeat compartments in multiple C57BL/6-derived ES cell lines. Our observations are consistent with previous studies highlighting epigenetic instability of methylation marks at imprinted loci in ES cells [25], [26]. It also supports the idea that the epigenetic state in C57BL/6 ES cells may be established early on following initial derivation and can be stably maintained over many passages. The fact that we observed hypomethylation in four out of eleven different derived lines suggests that C57BL/6-derived ES cell lines display greater epigenetic instability than 129/Sv-derived lines. We propose that this may be another contributing factor accounting for the lower germ-line transmission efficiencies observed when using ES cells derived from this genetic background [27]–[29].

Given the apparent normal methylation at IAP elements throughout the genome (with the obvious exception of clone B5 and its targeted derivatives), we asked whether the *A^vy^* IAP element is similarly normally methylated. Surprisingly, bisulphite sequencing of this IAP element in eight different cell lines revealed that the LTR sequence is essentially unmethylated (Figure 3C).

This provides an obvious explanation as to why 5-aza-treatment failed to induce *agouti/GFP* expression. Furthermore, although we were unable to directly assess the expression status of the *A^vy^* IAP element, these data suggest that at least in pluripotent stem cells, regulation of the downstream *agouti* locus is uncoupled from the methylation state of this IAP element's LTR.

To the best of our knowledge, this is the first study to examine the methylation state of the *A^vy^* IAP LTR in ES cells. Our initial intention was to generate a cell line that could be used in screen-based approaches to uncover novel epigenetic regulators of retrotransposon silencing. Our observation that the *A^vy^* IAP is apparently unmethylated offers insight into the unusual behaviour of the IAP at this particular locus. ES cells are derived from the inner cell mass of blastocyst embryos [30], [31]. Genome-wide loss of DNA methylation occurs following fertilization and prior to implantation of the blastocyst [32]. Indeed, such hypomethylation has previously been reported at the *A^vy^* locus from blastocysts derived from both pseudoagouti and yellow animals [33]. It is unclear whether the unmethylated state of the *A^vy^* IAP we observe in our various cell lines reflects a failure to correctly establish methylation following this erasure, or if methylation is correctly established but is rapidly lost upon ES cell derivation. Given that our bisulphite data indicate that methylation can (rarely) be detected at the locus, we favor the latter interpretation that the *A^vy^* IAP is inherently epigenetically unstable during the pluripotent state.

Genetic data have long supported the importance of DNA methylation in silencing IAP elements but the mechanism remains unclear [10], [20]. Two non-mutually exclusive models have been postulated [34]. The first is that the presence of methylation at LTRs prevents the binding of transcriptional activators. The second is that methyl binding proteins (MBPs) specifically recognize methylated CpGs within LTRs and directly recruit co-repressors to prevent gene expression.

Our results also indicate that the prevailing model of the *A^vy^* locus requires refinement. That is, control of the *A^vy^* IAP LTR cryptic promoter by DNA methylation and regulation of downstream *agouti* expression does not operate in pluripotent stem cells. Prior studies show that in pseudoagouti animals, where the *A^vy^* IAP is presumably silent, the IAP is significantly less methylated than other closely related IAPs [35]. Together with our observations, this indicates that the methylation state of the *A^vy^* locus does not control promoter activity of the *A^vy^* IAP. A broader implication of our study is that although IAP activity is constrained by DNA methylation it is likely that not all IAP elements are dependent on this mode of regulation. It has been estimated that there are between 1,000 to 2,500 IAP elements present per haploid genome [36]. Determining the elements controlled by DNA methylation remains technically challenging owing to the inherent repetitive nature of IAPs and unambiguously interrogating the activity of individual elements.

Through interference with transcription of nearby genes, retrotransposon-derived controlling elements have the potential to affect gene expression. The mechanisms by which they are silenced are not fully understood. Our results on the well-studied *A^vy^* locus demonstrate that DNA methylation-independent mechanisms ensure repression of the locus in pluripotent stem cells. A candidate pathway involves sequence specific KRAB-ZF proteins and KAP1, the latter being important in repression of endogenous retroviruses in murine ES cells [8]. Targeting of KAP1 to retroelements results in the recruitment of the histone methyltransferase SETDB1 [37] which is necessary for the addition of H3K9me3 marks at these elements which in turn result in the binding of HP-1 [38] and generates chromatin domains that are transcriptionally inert.

Finally, our data also suggests that the presence of DNA methylation at the *A^vy^* IAP in differentiated cells is a consequence of other signals that control the activity of the locus between generations. This has implications for the nascent field of environmental epigenetics and studies that attribute perturbations in DNA methylation, particularly in the early embryo, as the mechanism responsible for diet/environmentally induced alterations in gene expression.
